# Elevational variation in density dependence in a subtropical forest

**DOI:** 10.1002/ece3.1123

**Published:** 2014-05-30

**Authors:** Meng Xu, Shixiao Yu

**Affiliations:** Department of Ecology, School of Life Sciences/State Key Laboratory of Biocontrol, Sun Yat-sen UniversityGuangzhou, 510275, China

**Keywords:** Density dependence, Janzen–Connell hypothesis, mixed model, neighborhood effects, soilborne pathogens, subtropical forest

## Abstract

Density-dependent mortality has been recognized as an important mechanism that underpins tree species diversity, especially in tropical forests. However, few studies have attempted to explore how density dependence varies with spatial scale and even fewer have attempted to identify why there is scale-dependent differentiation. In this study, we explore the elevational variation in density dependence. Three 1-ha permanent plots were established at low and high elevations in the Heishiding subtropical forest, southern China. Using data from 1200 1 m^2^ seedling quadrats, comprising of 200 1 m^2^ quadrats located in each 1-ha plot, we examined the variation in density dependence between elevations using a generalized linear mixed model with crossed random effects. A greenhouse experiment also investigated the potential effects of the soil biota on density-dependent differentiation. Our results demonstrated that density-dependent seedling mortality can vary between elevations in subtropical forests. Species found at a lower elevation suffered stronger negative density dependence than those found at a higher elevation. The greenhouse experiment indicated that two species that commonly occur at both elevations suffered more from soilborne pathogens during seed germination and seedling growth when they grew at the lower elevation, which implied that soil pathogens may play a crucial role in density-dependent spatial variation.

## Introduction

Negative density dependence (NDD) has been recognized as a crucial mechanism that probably modulates tree abundance and maintains tree species diversity in forest communities (Wills et al. 1997; Webb and Peart 1999; Harms et al. 2000; Hubbell et al. 2001; Lambers et al. 2002; Wright 2002; Volkov et al. 2005; Chen et al. 2010; Comita et al. 2010; Metz et al. 2010; Zhu et al. 2010; Johnson et al. 2012). This spacing mechanism promotes species coexistence by preventing species from becoming disproportionately abundant at a local scale. For instance, a high-seedling density, close to a conspecific adult, due to the leptokurtic dispersal of seeds, may produce strong resource competition or be subject to pathogen infections. This results in higher mortality rates or a lower average growth rate, which leads to an increase in space for other species (Janzen 1970; Connell 1971; Wright 2002).

Previous studies have typically assessed NDD in a single community over a single time period (but see Lambers et al. 2002; Johnson et al. 2012; Lin et al. 2012; Piao et al. 2012; Shuai et al. 2014). However, because the two mechanisms that potentially drive NDD, that is, host-specific natural enemies and resource competition, are both influenced by environmental variation, the direction and strength of NDD may vary substantially in different environments (Coley and Barone 1996; Fajardo and McIntire 2011; Swinfield et al. 2012). For example, variation in NDD at different latitudes has been observed and is considered as a driving mechanism that may underpin regional tree species diversity patterns (Lambers et al. 2002; McCarthy-Neumann and Kobe 2010; Johnson et al. 2012; Shuai et al. 2014). Lin et al. (2012) have found that NDD differed between the dry and wet seasons in tropical forests. They attributed this variation to resource availability and to the natural enemy dynamics.

Variation in NDD due to a fluctuating environment would substantially influence species diversity. A stronger NDD can produce a stronger community compensatory trend (CCT) that depresses common species and provides opportunities for rare species, which promotes species diversity (Connell et al. 1984). If the per capita NDD is equal among species, abundant common species are expected to suffer higher overall mortality, which leads to a CCT (Webb and Peart 1999; Queenborough et al. 2007; Chen et al. 2010). However, per capita NDD may vary among species. For example, negative conspecific effects have been found to decrease as the relative abundance of a species increases, a pattern that appears to contradict a possible CCT (Comita et al. 2010; Lewis 2010; Mangan et al. 2010; Johnson et al. 2012). In this situation, a CCT still results if the local conspecific neighbor densities increase with species abundance (Kobe and Vriesendorp 2011). Lin et al. (2012) have verified this hypothesis in the Xishuangbanna tropical forest. They found that although rare species suffered stronger negative effects per individual neighbor, individuals of common species experienced higher local conspecific densities at the community level, which resulted in a declining seedling survival rate as the population abundance rose. Moreover, theoretical studies have also indicated that rare species suffering from stronger conspecific negative effects can persist in a community and that diversity can be maintained (Chisholm and Muller-Landau 2011; Yenni et al. 2012). Nevertheless, NDD has substantial impacts on community composition and species diversity. Therefore, investigating NDD variation will improve our understanding of how forest community diversity is maintained.

An important aspect of our investigation into NDD variation was a study of the underlying mechanisms. As mentioned previously, the natural enemies and resource competition dynamics can be affected by environmental factors and may produce variations in NDD. For example, forest communities experience distinct elevational variations in temperature and humidity. The higher temperatures and humidities at low elevations may increase the activity and density of pathogens (Burdon 1987), thereby contributing to a stronger NDD. Alternatively, species may be under greater stress at high elevations due to the low temperature and humidity. Hence, it is more probable that they will die as a result of density dependence. In addition, many studies have indicated that under extremely stressful conditions, competition may become weaker and facilitation may occur (Callaway 2007; Anderson 2009; Fajardo and McIntire 2011).

The seedling stage represents a bottleneck in the life cycle of trees (Harper 1977), which has meant that it has become the focus of research into NDD. However, competition between seedlings has been shown to be unimportant for seedling recruitment (Paine et al. 2008; Svenning et al. 2008). Over the past few decades, the biotic mechanisms that underlie NDD patterns have been widely studied. The Janzen–Connell (J–C) hypothesis (Janzen 1970; Connell 1971) is considered to be a leading mechanism behind the maintenance of diversity in tropical forests and has received considerable empirical support (see reviews by Freckleton and Lewis 2006; Carson et al. 2008; Mordecai 2011). The J–C hypothesis proposes that the offspring survival rate should increase as the distance from conspecific adults increases and conspecific offspring density should decrease due to predation by specialized natural enemies (pathogens and herbivores). This favors the establishment of heterospecific offspring near the adults and results in increased species diversity. Therefore, host-specific pests, such as pathogens and herbivores, may be the main agents driving NDD and higher species diversity (Bagchi et al. 2014).

It is reasonable to expect that pathogens are the principal driver of NDD at the seedling stage. Indeed, soilborne fungal pathogens that cause damping-off diseases in young seedlings have been shown to cause density and/or distance-dependent seedling mortality (Augspurger 1983; Augspurger and Kelly 1984; Packer and Clay 2000; Hood et al. 2004; Bell, Freckleton and Lewis 2006; Reinhart and Clay 2009; Bagchi et al. 2010; Hersh et al. 2012; Liu et al. 2012a,b2012b). It has also been demonstrated that soilborne pathogens influence the relationship between plant diversity and productivity (Maron et al. 2011; Schnitzer et al. 2011), if it is assumed that the rate of host-specific pathogen transmission decreases as diversity increases. However, future research needs to investigate whether soilborne pathogens cause the NDD differentiation observed in natural tree communities.

In this study, we report observational and experimental results. We first tested for spatial variation in the effects of local conspecific and heterospecific neighbors on seedling survival in a subtropical evergreen broadleaved forest. We examined elevational differences in density dependence using survival data from 5216 seedlings of 112 species at two elevations. Then, we experimentally tested for the effects of soilborne pathogens on the seedling survival of species growing in soil collected from beneath the adults of the same species at low and high elevations. We found that the species suffered strong conspecific seedling NDD at the low-elevation site, whereas NDD was absent at the high-elevation site. Soilborne pathogens exerted a stronger negative effect on species at the low-elevation site and may play a crucial role in NDD elevational variation in subtropical forests.

## Materials and Methods

### Study sites

The field work was conducted at Heishiding Natural Reserve (111°53′E, 23°27′N, 150–927 m above sea level), in Guangdong Province, China. The reserve covers around 4200 ha of subtropical evergreen broad-leaved forest and the Tropic of Cancer runs through its center. The region has a subtropical moist monsoon climate. The mean annual temperature is 19.6°C, with mean monthly temperatures ranging from 10.6°C in January to 28.4°C in July. The annual precipitation averages 1744 mm, with a humid season from April to September and a dry season from October to March. The dominant tree species in this region belong to the Fagaceae and Lauraceae families, which are broadly distributed in subtropical evergreen broadleaved forests (Yu et al. 2000).

### Field surveys

Six 1-ha permanent plots were established and observed from winter 2007 until spring 2008 to estimate the effect of neighboring tree species on seedling recruitment in the natural community. Three of the plots were located at a relatively high elevation (600 m above sea level) and three plots were located at a low elevation (340 m). All three plots at each altitude were close to one another (two of the low-altitude plots are adjacent) and the horizontal distance between the high-altitude plots and the low-altitude plots was about 300 m. All saplings and adult tree stems ≥1 cm diameter at breast height (DBH) in the plots were tagged, measured, mapped, and identified to the species level (Liu et al. 2012a). In all, 181 species from 106 genera and 55 families were identified, comprising approximately 29,800 individuals. A previous study of the area indicated that species composition did not change significantly with elevation (Liu et al. 2012a).

In spring 2008, we established 300 census stations, which were used to monitor seedling dynamics in the plots. Fifty census stations were located within each 1-ha plot. Each station consisted of one 1 m^2^ seed trap and four 1 m^2^ seedling quadrats (Fig. S1). In each of the 1200 seedling quadrats, all woody plant seedlings with a DBH <1 cm were tagged, measured, and identified to the species level. The census was repeated in spring 2009. We also collected seedling mortality data during the second census.

### Greenhouse experiment

We examined the effects of soilborne pathogens on seed germination and seedling growth using two species (*Engelhardia fenzelii* and *Cryptocarya concinna*) that commonly occurred in both the low- and high-elevation field sites. These species were chosen because the two species were dominant in the Heishiding Nature Reserve plant communities and because the seeds could be stored and rapidly germinated after surface sterilization. The seeds were collected from the low-elevation site during autumn and winter 2010. They were surface sterilized (0.5% KMnO_4_ for 2 h), rinsed with water three times, and then kept in a refrigerator at 4°C. The seeds were soaked in water for 24 h before planting.

In January 2011, four adult trees of each species in the low- and high-elevation plots were chosen randomly as inoculum sources. We collected soil at a depth of 0–20 cm and at a distance of 0–1 m away from the adult trees. Soil samples from the same species and elevation were mixed to create composite samples. They were then transported to the laboratory in Guangzhou and sieved to eliminate any seeds and stones. The soil samples from each species at the low- or high-elevation sites were divided into two parts. One part was left untreated (the control), whereas the second part was autoclaved for 2 h at 121°C (sterilized treatment). These soil samples were then placed in pots that were 14 cm in diameter and 12 cm in height. The bottom half of the pot was filled with sterilized sand. A total of 500 g of either untreated or sterilized soil was then added to the pots. Six seeds of *Cryptocarya concinna* and 15 seeds of *Engelhardia fenzelii* were planted in the pots containing their parent soil. These pots were placed in two greenhouses in which the field conditions at the low and high elevations were reproduced. Specifically, the pots containing soil samples from the low-elevation site were placed in a greenhouse that was kept at 27°C, and the soil humidity was maintained at 30% by controlling the watering frequency. The pots containing the soil samples from the high-elevation site were maintained at a temperature of 21°C and at a soil humidity of 15%.

Collectively, four treatment combinations (two elevations × two soil treatments) were used for each species. Each treatment combination was replicated 10 times, and the positions of the pots in the greenhouses were randomly changed every week. The experiment lasted for 4 months and seed germination and seedling growth were determined every 2 weeks.

### Statistical analysis

We modeled the survival probability as a function of local neighborhood variables using generalized linear mixed models (GLMMs) and assumed that there was a binomial error and logit-link function (Bolker et al. 2009). We used the “lme4” package and MCMC sampler to calculate the *P*-values (Baayen et al. 2008; Bates et al. 2012). We included local neighborhood density variables and elevation as fixed effects when species identity, seedling quadrat, station, and plot were entered as random effects. The quadrat-specific and station-specific random effects were used to incorporate potential spatial autocorrelation in seedling survival within the same quadrat and station (Dormann et al. 2007). A plot-specific random effect was used to characterize the variation between plots. We also included species identity as a crossed random effect because species with different ecological strategies were expected to respond differently to neighborhood variables (Lin et al. 2012). This design allowed the species used in the study to be viewed as a random sample of all the species in the community and provided a convenient framework for comparing density dependence between elevations (Chen et al. 2010). We also included initial seedling height as a covariate in the models because seedling height is a significant predictor of survival. The GLMM for seedling survival with crossed random effects is as follows:


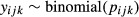



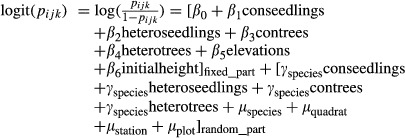


where *y*_ijk_ is the binary response (alive or dead) for seedling *i* in plot *j* and station *k* and *p*_ijk_ is the predicted survival probability for each seedling. In the fixed part of the model, *β*_0_ denotes the fixed intercept. *β*_1_, *β*_2_, *β*_3_, *β*_4_, *β*_5,_ and *β*_6_ represent the mean coefficient values across all species for conspecific seedling abundance, heterospecific seedling abundance, conspecific tree abundance, heterospecific tree abundance, elevation and initial seedling height, respectively. *γ*_species_ denotes the random slope for each species (i.e., the four parameters for local neighborhood density vary among species). *μ*_species_ characterizes the variation among species and *μ*_quadrat_, *μ*_station,_ and *μ*_plot_ describe the survival autocorrelation within the seedling quadrat, station, and variation among 1 ha plots, respectively.

The local neighborhood variables used in this study included conspecific and heterospecific seedling density in a 1 m^2^ quadrat, the abundance of conspecific and heterospecific trees and the basal area of conspecific and heterospecific trees (DBH ≥ 1 cm). For each seedling, we summed the number of conspecific or heterospecific seedling neighbors that existed in the seedling quadrat during the census. We also summed the conspecific or heterospecific tree abundance and the conspecific or heterospecific tree basal area within a 20 m radius from each station's seed trap. The 20 m cutoff was selected because previous analyses have shown that neighbor effects are not usually significant beyond 20 m (Hubbell et al. 2001).

We also defined another GLMM to test the effects of relative conspecific seedling density, relative abundance of conspecific trees and relative basal area of conspecific trees on seedling survival. In this model, we included the proportion of conspecific seedlings in a 1 m^2^ quadrat, the conspecific tree abundance proportion, the conspecific tree basal area proportion within a 20 m radius and elevation as fixed effects and the initial seedling height as a covariate. The random part of this model was the same as that of the first model. The relative measurements were chosen as predictors because the variation in the relative value produced a better characterization of species coexistence (Adler et al. 2007; Siepielski and McPeek 2010).

We performed a model selection procedure and compared the models containing the different fixed effects mentioned above (Table 1). The best-fit model was selected as the one with the lowest Akaike information criterion (AIC). We also examined the data from the low- and the high-elevation sites separately. For both elevations, we performed the model comparison and selected the best model to analyze the effects of neighbors.

**Table 1:** Comparison of generalized linear mixed models of seedling survival in the six 1-ha plots

Models	Fixed effects	AIC	BIC	logLink
1	Ht + Cons + Hets + Cona + Heta	1702.6	1832.2	−829.3
2	Ht + Cons + Hets + Conb + Hetb	1681.8	1811.4	−818.9
3	Ht + Cons + Hets + Cona + Heta + Ele	1692.4	1827.9	−823.2
4	Ht + Cons + Hets + Conb + Hetb + Ele	**1672.7**	**1808.2**	−**813.3**
5	Ht + Cons + Hets + Cona + Heta + Conb + Hetb + Ele	1694.8	1842.0	−822.4
6	Ht + Cons + Hets + Cona + Heta + Ele + Cons:Ele + Cona:Ele	1692.8	1840.1	−821.4
7	Ht + Cons + Hets + Conb + Hetb + Ele + Cons:Ele + Conb:Ele	1676.7	1823.9	−813.3
8	Ht + Rel-Cons + Rel-Cona	1666.2	1731.0	−822.1
9	Ht + Rel-Cons + Rel-Conb	1671.0	1735.8	−824.5
10	Ht + Rel-Cons + Rel-Cona + Ele	**1652.6**	**1723.2**	−**814.3**
11	Ht + Rel-Cons + Rel-Conb + Ele	1655.8	1726.5	−815.9
12	Ht + Rel-Cons + Rel-Cona + Rel-Conb + Ele	1660.7	1760.9	−813.4
13	Ht + Rel-Cons + Rel-Cona + Ele + Rel-Cons:Ele + Rel-Cona:Ele	1653.9	1736.3	−812.9
14	Ht + Rel-Cons + Rel-Conb + Ele + Rel-Cons:Ele + Rel-Conb:Ele	1656.6	1739.0	−814.3

Ht, initial seedling height; Cons, conspecific seedling density; Hets, heterospecific seedling density; Cona, conspecific tree abundance; Heta, heterospecific tree abundance; Ele, elevation; Conb, conspecific tree basal area; Hetb, heterospecific tree basal area; Rel-Cons, relative conspecific seedling density; Rel-Cona, relative conspecific tree abundance; Rel-Conb, relative conspecific tree basal area.

Significant results are shown in boldface type.

For the greenhouse experiment, we calculated the relative soil biota effect based on seed germination (log seed germination rate in the control soil – log seed germination rate in the sterilized soil) and seedling growth (log seedling height in the control soil – log seedling height in the sterilized soil) at the low and high elevations. A two-way ANOVA was used to test the effects of sterilization, elevation and their interaction. A Student's *t*-test was used to compare the relative soil biota effects between the low and the high elevations and to test whether the mean of the relative soil biota effects differed significantly from 0. All statistical analyses were performed using R 2.15.0 (R Development Core Team 2011).

## Results

### Field surveys

**Figure 1:**
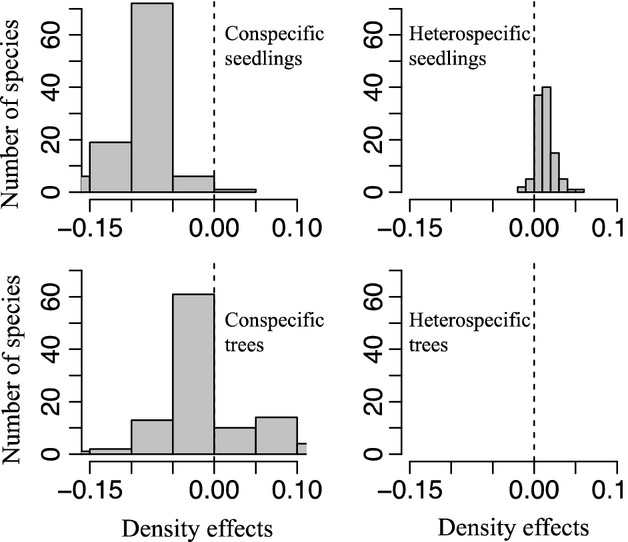
Distribution of effects of conspecific and heterospecific neighbors on seedling survival in 6 1-ha plots.

The best model for absolute neighbor effects used conspecific seedling density, heterospecific seedling density, conspecific tree basal area, and heterospecific tree basal area as the fixed effects. The best model for relative neighbor effects used the relative conspecific seedling density and relative conspecific tree abundance as the fixed effects (Table 1). In both models, elevation was an important variable in explaining seedling survival (Table 1), with low-elevation trees having a significantly lower survival rate than high-elevation trees (Table 2). Conspecific seedling neighbors had a significant negative effect on seedling survival (Table 2) at the six 1-ha sites. The strength of the relative conspecific seedling effects varied widely for the two species but was mostly negative (Fig. 1). In contrast, the effects of heterospecific seedlings and heterospecific tree neighbors were close to zero for the two species. The conspecific tree effects varied considerably for both species but the average value was close to zero (Table 2, Fig. 1). The relative conspecific seedlings effects had a significant negative impact on the seedling survival, whereas relative conspecific tree effects did not significantly affect seedling survival (Table 2).

**Table 2:** Parameter estimates and significance in generalized linear mixed models (model 4 and model 10) that predicted neighbor effects of seedlings on seedling survival in six 1-ha plots

Fixed effect	Estimate	*Z*-value	*P* (>|z|)
Initial height of seedlings	0.6856	7.114	**<0.0001**
Conspecific seedlings	−0.0935	−2.480	**0.0131**
Heterospecific seedlings	0.0115	0.461	0.6447
Conspecific trees	−0.0127	−0.313	0.7546
Heterospecific trees	0.2694	1.256	0.2091
Elevations	0.6737	−3.507	**0.0005**
Initial height of seedlings	0.7110	7.373	**<0.0001**
Relative conspecific seedlings	−0.7031	−2.517	**0.0118**
Relative conspecific trees	−0.4886	−0.423	0.6722
Elevations	0.8337	−4.036	**<0.0001**

Significant results are shown in boldface type.

At both low and high elevations, the best models of the absolute neighbor effect contained the conspecific seedling density and heterospecific seedling density as fixed effects. At the low elevation, the best model of the relative neighbor effect contained the relative conspecific seedling density and relative conspecific tree abundance as fixed effects, whereas at the high elevation, the best fixed effects were the relative conspecific seedling density and the relative conspecific tree basal area. For the low-elevation plant community, both the conspecific seedling density and the relative conspecific seedling density had significant negative effects on seedling survival (Table 3). In contrast, heterospecific seedlings and relative conspecific tree abundance had no significant effects on seedling survival (Table 3). However, none of these species-specific neighbor effects were significantly correlated with seedling survival at the high-elevation sites (Table 3).

**Table 3:** Parameter estimates and significance in generalized linear mixed best-fit models that predicted neighbor and relative neighbor effects on seedling survival in low- and high-elevation plots

	Parameter estimate			
	
	Low elevation		High elevation	
		
Fixed effect	Estimate	Pr (>|z|)	Estimate	Pr(>|z|)
Initial height of seedlings	0.6992	**<0.0001**	0.6430	**0.0018**
Conspecific seedlings	−0.0337	**0.0472**	−0.0578	0.1603
Heterospecific seedlings	0.0030	0.9098	0.0712	0.3698
Initial height of seedlings	0.7498	**<0.0001**	0.6670	**0.0014**
Relative conspecific seedlings	−1.0711	**0.0157**	−0.4067	0.4361
Relative conspecific trees	1.6633	0.3908	0.3168	0.9104

Significant results are shown in boldface type.

### Greenhouse experiments

**Figure 2:**
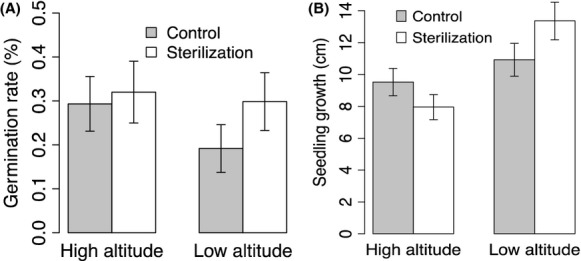
Greenhouse experiments testing the effect of elevations (low vs. high) and soil treatments (sterilization vs. control) on seed germination (A) and seedling growth (B). Bars represent means ± 1 SE.

**Figure 3:**
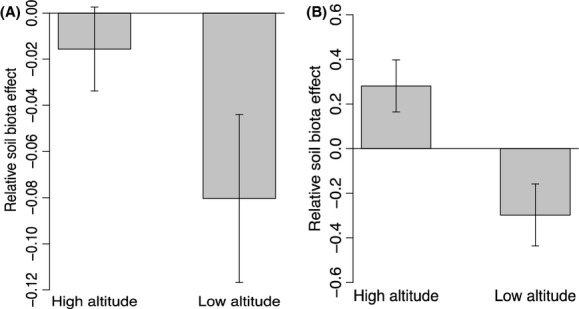
Greenhouse experiments testing the effect of elevation on the relative soil biota effect based on seed germination (A) and seedling growth (B). Bars represent means ± 1 SE.

In the greenhouse experiment, we found that soil biota had a negative effect on seed germination and seedling growth for both species in the low-elevation experiment. We have reported the combined results for these two species because the effects of soil sterilization and elevation on seed germination and seedling growth were highly consistent for the two species and because we were more interested in overall community responses rather than species-specific results. Sterilization (ANOVA, *F* = 0.99, *P* = 0.322), elevation (*F* = 0.92, *P* = 0.341), and sterilization × elevation (*F* = 0.45, *P* = 0.503) did not significantly influence seed germination (Fig. 2A). However, the low-elevation simulation experiment showed increased seedling growth compared with the high-elevation simulation experiment (*F* = 10.99, *P* = 0.002, Fig. 2B), and this elevation effect was affected by soil sterilization (*F* = 3.21, *P* = 0.080, Fig. 2B). Even though there was no significant difference between elevations for the relative soil biota effect on the seed germination rate (*t*-test, *t* = 1.59, *P* = 0.123, Fig. 3A), soil biota significantly affected the plants in the low-elevation simulation experiment (*t* = −2.20, *P* = 0.040, Fig. 3A) compared with the high-elevation experiment (*t* = −0.86, *P* = 0.402, Fig. 3A). The relative soil biota effects on seedling growth differed significantly between elevations (*t* = 3.18, *P* = 0.005, Fig. 3B). In the low-elevation simulation experiment the soil biota depressed seedling growth (*t* = −2.14, *P* = 0.061, Fig. 3B), whereas soil biota in the high-elevation experiment had a positive effect (*t* = 2.40, *P* = 0.037).

## Discussion

In this study, we examined the data from a field survey to explore the variation in density dependence between two different elevations in a subtropical forest. We also investigated the potential role of the soilborne biota in driving this elevational differentiation. Our results indicated that at the low-elevation sites, the strong negative effects of conspecific seedlings limited seedling survival, whereas conspecific and heterospecific neighbors had no significant effects on seedling survival in the high-elevation sites. The negative effects of soil pathogens on seed germination and seedling growth were stronger in the low-elevation sites than in the high-elevation sites. This suggested that soilborne pathogens played an important role in density dependence differentiation in subtropical forests.

Conspecific NDD appeared to be stronger in the low-elevation sites than in the high-elevation sites. This result was consistent with the strong negative effect of relative conspecific seedlings in the low-elevation sites. Spatial variation, together with the changes in environmental conditions, may alter the local interaction between plant species and host-pathogen interactions, which would affect the strength of the NDD (Benitez et al. 2013). The stress gradients hypothesis predicts that facilitative interactions will become more important when stress or consumer pressure are high (Maestre et al. 2009; Malkinson and Tielbörger 2010; Fajardo and McIntire 2011). As elevation increases, temperature and soil moisture declines and understory light availability rises. These changes may directly influence the interactions between plants, possibly making facilitation more important than competition at high elevations, which would buffer NDD changes. For example, Bunker and Carson (2005) found that increased soil moisture favored NDD in seasonal forests in Panama. In our study, the lapse rate related to the change in elevation between the high and low-elevation sites (about 300 m) was about 1.5°C. If we combine this with the anticipated declines in air and soil moisture at high-elevation sites, compared with low-elevation sites, we should expect lower competition for resources between plant species at the high-elevation sites. However, the change in environmental conditions may regulate host-pathogen interactions (Burdon and Thrall 2009; Tack et al. 2012). Changes in environmental conditions can affect host and pathogen physiological statuses, as well as dispersal and establishment (Pariaud et al. 2009; Laine et al. 2011). Several studies have investigated how light availability and soil moisture affects survival and disease development (Ichihara and Yamaji 2009; Alvarez-Loayza et al. 2011; Hersh et al. 2012). For example, soil moisture has been found to affect the co-infection combination (Hersh et al. 2012). Light intensity variations changed endosymbiotic fungi into pathogens and this affected seedling survival (Alvarez-Loayza et al. 2011). Thus, higher temperatures and moistures and lower understory light availability at lower elevations may increase pathogen activity, and this could result in a higher risk of disease and a stronger NDD effect.

Two studies that compared density dependence between a tropical forest and a temperate forest produced mixed results (Lambers et al. 2002; McCarthy-Neumann and Kobe 2010). McCarthy-Neumann and Kobe (2010) showed that tropical tree species suffered NDD more frequently than temperate tree species, whereas Lambers et al. (2002) concluded that the proportion of species affected in temperate forests was equivalent to that in tropical forests. However, they investigated the variation in density dependence between latitudes in terms of the frequency of species affected by NDD rather than in terms of the NDD strength. Our study focused on the variation in the strength of NDD between elevations and stressed the effect of environmental factors on soilborne pathogens. Our results support the hypothesis that the NDD mechanism is stronger in a forest community with high temperatures and high soil moistures, which are the conditions found in a tropical forest (Shuai et al. 2014).

In contrast to the effects caused by conspecific seedling neighbors, conspecific tree neighbors and heterospecific neighbors had no significant effects on seedling survival at the low- or the high-elevation sites. These results, especially for conspecific tree neighbors, were in contrast to several recent studies that have indicated that conspecific trees had strong negative effects on seedling survival (Chen et al. 2010; Comita et al. 2010; Metz et al. 2010; Lin et al. 2012). The Janzen–Connell hypothesis proposes that species-specific enemies are more prevalent near adult trees. This factor could be the potential reason for the negative effects caused by conspecific trees. However, conspecific trees may also provide an appropriate local habitat for conspecific seedling growth, which would mitigate the negative effects caused by attacks from natural enemies. In addition, several studies have also shown positive effects by heterospecific neighbors (Peters 2003; Chen et al. 2010; Lin et al. 2012), which supports the herd protection hypothesis (Wills and Green 1995). The demographic analysis carried out in our study did not detect this phenomenon in the low- or the high-elevation sites.

We found wide variation among species in the effect of conspecific seedlings on survival. However, in contrast to the results of a recent study (Comita et al. 2010), we did not find a significant relationship between the strength of the conspecific neighbor effects and species abundance (Fig. S2). Although the association between NDD and species abundance has recently received some empirical and theoretical support (Mangan et al. 2010; Chisholm and Muller-Landau 2011; Kobe and Vriesendorp 2011; Johnson et al. 2012), there should be more research into whether other species characteristics, such as shade tolerance (Kobe 1999; McCarthy-Neumann and Kobe 2008; Comita and Hubbell 2009), also affect NDD variation among species. Unfortunately, we did not quantify the value of other species traits and therefore cannot examine their potential role in driving the NDD variation. Future studies should consider species characteristics in other forests and examine the specific mechanisms that underlie the relationship between NDD and species abundance. This would improve our understanding of species composition and diversity maintenance.

We conducted a manipulative experiment to assess the mechanisms that underpin elevational variation in NDD. Although studies testing the potential mechanisms behind NDD have often examined the role of soil-borne pathogens (Augspurger and Kelly 1984; Packer and Clay 2000; Bell et al. 2006; Bagchi et al. 2010; Reinhart et al. 2010; Liu et al. 2012b), few experiments have attempted to explore how they drive NDD temporal and spatial variation (McCarthy-Neumann and Kobe 2010; Shuai et al. 2014). A recent study that investigated seasonal differences in density dependence found that NDD was stronger in the dry season than in the wet season, which indicated that a decrease in soil water resources influenced the negative neighborhood effect (Lin et al. 2012). The study results suggested that this was due to a greater susceptibility to natural enemies and/or aggravated competition in the dry season. Alternatively, the increased abundance, activity and transmission of soil-borne pathogens at high temperatures and high moisture levels may intensify the NDD strength (Wong et al. 1984; Coley and Barone 1996; O Hanlon-Manners and Kotanen 2004; Swinfield et al. 2012), which would explain the stronger NDD observed in the low-elevation sites. The increased negative feedback in the simulated low-elevation environment confirmed our speculation that increased soil temperature and moisture affected the abundance and/or activity of soil pathogens, thus inducing a stronger NDD. However, the origin of the seeds may potentially influence the results of the greenhouse experiment. Parental effects are known to influence plant responses and plasticity in the next generation. All the seeds used in the greenhouse experiment were collected from the low-elevation site, which meant that they were probably adapted to a low-elevation environment. In this case, we would expect a better performance from the seedlings that had been grown in the low-elevation site simulation experiment. Indeed, we found that seedling growth was greater in the low-elevation site experiment than in the high-elevation site simulation experiment, whereas there was no difference in the germination rates between the two elevations. Adaption to the parental environment may make seeds more resistant to soil biota attack if the soil biota effects were the same at the low and high-elevation sites. However, there was a stronger negative feedback in the low-elevation experiment, which suggested that there was a stronger soil biota negative effect at low elevation.

In this study, we aimed to demonstrate the treatment effects at the community level and did not attempt to clarify details about effects caused by individual adults. So, in the greenhouse experiment, we bulked soil samples taken from the soil surrounding four adult plants. However, some studies have shown that the identity of the adult is important (Reinhart and Clay 2009), so our results may have over inflated the importance of the strong negative soil biota effects at one adult tree out of the four sampled.

Autoclaving the soil also eliminated other soil biota, such as arbuscular mycorrhizal fungi (AMF), which may have led to a nutrient flush (Packer and Clay 2000). If AMF played a more important role than soilborne pathogens, then sterilization would have decreased seed germination or seedling growth relative to the unsterilized treatments. The opposite effect would suggest that soil-borne pathogens decreased seed germination. Nevertheless, we found a positive soil biota effect on seedling growth in the simulated high-elevation environment, which highlighted the complex interactions between the soil biota and the abiotic environment. Additional studies are required to identify the different responses by soil organisms to changes in environmental conditions and to understand the plant community and species diversity implications of these differences.

## Conclusions

Our results suggest that local scale NDD tends to be stronger at low elevations than at high elevations in the subtropical forest and that soilborne pathogens play a crucial role in this elevational variation in NDD. Our study also highlights the need to incorporate NDD spatial variation into future theoretical and empirical efforts to identify the true characteristics of the natural community. Combining the seasonal and latitudinal variation in NDD found by recent studies (Johnson et al. 2012; Lin et al. 2012; Shuai et al. 2014), suggests that the temporal and spatial variation in NDD may strongly influence the species coexistence and diversity patterns in forest communities. Further research is also required to identify specific soil-borne pathogens and test their host specificity to improve understanding of the processes underlying species diversity patterns and to simultaneously consider the influence of other environmental factors, such as elevated CO_2_ concentrations, on soil biota effects.

## Supporting Information

**Figure S1.** Details of seedling quadrat network design. **Figure S2.** The mean strength of conspecific NDD exhibited by individual tree species (points) is not related to species abundance in the low-elevation (A) and high-elevation communities (B) of the subtropical forest.
