# Supplementary material for: Elevational variation in density dependence in a subtropical forest
Source: Ecol Evol. 2014 May 30;4(14):2823–33. doi: 10.1002/ece3.1123 (PMC4130442; doi:10.1002/ece3.1123)
Supplement: Supplementary file 1 [file ece30004-2823-SD1.docx]

**Supporting information**

**Fig. S1** Details of seedling quadrat network design. We established six 1-ha permanent plots during winter 2007 to spring 2008. Three of them are located at relatively high altitude (600 m above sea level) and the other three at low altitude (340 m). All three plots at each altitude are close to one another (two of the low altitude plots are adjacent), and the horizontal distance between the high altitude plots and the low altitude plots is about 300 m. Fifty census stations are regularly located within each 1-ha plot. Each station includes one 1-m^2^ seed trap and four 1-m^2^ seedling quadrats. Within each seedling quadrat, all seedlings of woody plants with DBH < 1 cm are mapped, tagged, identified to species, and their height measured in spring 2008.

**Fig. S2** The mean strength of conspecific NDD exhibited by individual tree species (points) is not related to species abundance in the low-elevation (a) and high-elevation communities (b) of the subtropical forest.
